# PROJECT, PERSUADE, AND PROTECT: ADULT CHILDREN’S PREDICTION OF PARENTS’ HEALTH STATE VALUATIONS

**DOI:** 10.1093/geroni/igad104.3538

**Published:** 2023-12-21

**Authors:** Jessica Hahne, Rachel E Buenger, Tim Chung, Hannah Silverstein, Brian Carpenter

**Affiliations:** Washington University in St. Louis, St. Louis, Missouri, United States; Washington University in St. Louis, St. Louis, Missouri, United States; Washington University in St. Louis, St. Louis, Missouri, United States; Washington University in St. Louis, St. Louis, Missouri, United States; Washington University in St. Louis, St. Louis, Missouri, United States

## Abstract

Serious illness decision making for older adults frequently involves their adult children. Thus, it is important that adult children are familiar with parents’ illness preferences. In this mixed methods analysis (total N = 114), we examined concordance between older adults’ valuations of illness states, their children’s predictions of their valuations, reasoning behind children’s predictions, and children’s reactions to discordance between their predictions and parents’ valuations. Thirty-eight older parents (M age= 75.8) provided valuations for five different illness states. Two adult children (M age= 46.0) independently predicted their parent’s answers. Family triads participated in a semi-structured conversation about their responses. One-way random effects, absolute agreement, average measures intraclass correlation coefficients (ICCs) showed that average concordance within families was poor for incontinence (ICC = .172, 95% CI [-.497, .572]), dialysis (ICC = .300, 95% CI [-.264, .639]), tube feeding (ICC = .320, 95% CI [-.228, .649]), and chronic pain (ICC = .438, 95% CI [-.015, .710]), and moderate for Alzheimer disease (ICC = .556, 95% CI [.198,.771]). Results from qualitative thematic analysis indicated that children often projected their own values when attempting to predict parents’ valuations. When faced with discordance, many children attempted to persuade parents to change their valuations, while also making assertions to protect the parent’s autonomy. In summary, adult children navigating surrogate or shared decision making may face challenges inferring parent values versus their own and balancing their input with parent autonomy. Future research and interventions in late-life families could target these challenges.

